# Significant survival benefit of adjuvant chemotherapy after concurrent chemoradiotherapy in locally advanced high-risk nasopharyngeal carcinoma

**DOI:** 10.1038/srep41449

**Published:** 2017-02-02

**Authors:** Zhong-Guo Liang, Xiao-Qian Chen, Guo-Xiang Lin, Bin-Bin Yu, Kai-Hua Chen, Qiu-Lu Zhong, Si-Kai Nong, Ling Li, Song Qu, Fang Su, Wei Zhao, Ye Li, Xiao-Dong Zhu

**Affiliations:** 1 Department of Radiation Oncology, The Affiliated Tumour Hospital of Guangxi Medical University, Cancer Institute of Guangxi Zhuang Autonomous Region, 71 He Di Road, Nanning 530021, P.R. China

## Abstract

The present study aimed to define high-risk patients who may benefit from additional adjuvant chemotherapy (AC) after concurrent chemotherapy in combination with intensity-modulated radiotherapy among patients with loco-regionally advanced nasopharyngeal carcinoma (NPC). A cohort of 511 NPC patients who received concomitant chemoradiotherapy (CCRT) with or without AC between January 2007 and December 2012 were retrospectively analysed. One hundred seventy-seven patients received CCRT alone, whereas 334 received CCRT + AC. The survival analysis showed that ages >45 years old, T3-T4 stages, N2-N3 disease and serum albumin levels ≤42 g/L were significant independent prognostic factors for overall survival (OS). Using these four risk factors, a prognostic model for OS was created as follows: (1) low-risk group: 0–1 risk factors; and (2) high-risk group: 2–4 risk factors. In the CCRT alone and CCRT + AC groups, significant differences in survival were found between the high- and low-risk groups. Patients in the high-risk group exhibited improved OS due to the addition of AC to CCRT, but no survival benefits were found in the low-risk group. In conclusion, high-risk patients may benefit from the addition of AC to CCRT regarding OS.

According to a survey from the International Agency for Research on Cancer, an estimated 86,700 new cases of nasopharyngeal carcinoma (NPC) and 50,800 deaths occurred in 2012. The incidence rates are highest in Southeast Asia, including Malaysia, Indonesia, and Singapore, and in Southeast China^1^. The National Comprehensive Cancer Network guidelines (version 1, 2016) recommend the use of concurrent chemoradiotherapy (CCRT) followed by adjuvant chemotherapy (AC) as the standard treatment for loco-regionally advanced NPC (Category 2 A). However, controversy exists regarding whether NPC patients can benefit from additional AC after chemotherapy in combination with intensity-modulated radiotherapy (IMRT).

Several randomized controlled trials (RCTs)^2^,^3^,^4^ have been conducted to evaluate the value of AC after CCRT for loco-regionally advanced NPC. In two RCTs^2^,^3^, the concurrent chemotherapy regimen was cisplatin alone, while uracil and tegafur were used in a trial by Kwong *et al*.^4^. In these three RCTs^2^,^3^,^4^, the AC regimens all consisted of cisplatin and 5-fluorouracil. None of these trials showed survival benefits with the addition of AC to CCRT. In 2015, Yan *et al*.^5^ and Chen *et al*.^6^ each performed a Bayesian network meta-analysis to assess chemoradiotherapy regimens for loco-regionally advanced NPC. Neither study found a significant difference in survival between patients who received CCRT + AC or CCRT alone. However, the additional AC may benefit certain patients with NPC. In a trial conducted by Xu *et al*.^7^, the efficacies of different chemotherapy sequences with radiotherapy were compared in patients with stage N3 disease; cisplatin alone was used as the concomitant chemotherapy regimen, and the combination of cisplatin with 5-fluorouracil was utilised as the AC regimen. They found that patients in the CCRT + AC group exhibited significantly higher 5-year overall survival (OS) and distant metastasis failure-free survival (DMFS) rates than those in the CCRT-alone group. Therefore, the addition of AC after CCRT may be essential for treating N3 stage NPC.

In the era of precision medicine, individualised treatment regimens have become increasingly urgent. To further determine who may gain survival benefits from the addition of AC to CCRT, we retrospectively analysed a cohort of patients to assess the independent prognostic factors and then constructed a prognostic score model.

## Methods

### Patients

A cohort of 523 NPC patients treated between January 2007 and December 2012 were retrospectively included. These patients were all newly diagnosed and pathologically proven to have NPC without distant metastases. They received IMRT and concurrent chemotherapy with or without AC. Because of the partial data loss of 12 patients, data from 511 patients were ultimately analysed. One hundred seventy-seven patients received CCRT alone, whereas 334 received CCRT + AC. Three hundred ninety-five patients were male, and 116 were female. The details of the two groups are shown in Table 1. The Ethics Committee of the Affiliated Tumour Hospital of Guangxi Medical University approved the study protocol, and all patients provided signed informed consent.

### Treatment strategies

A detailed description of IMRT has been published previously^8^. Nasopharynx gross tumour volume (GTVnx) included the gross tumour in the nasopharynx, and the gross tumour volume of the neck lymph nodes (GTVnd) included positive lymph node areas. A high-risk clinical tumour volume (CTV1) included the GTVnx with a 5–10 mm margin (forward, both sides, top and bottom) and a 3–5 mm margin (back). A low-risk clinical tumour volume (CTV2) included the GTVnd and lymphatic regions based on the tumour invasion pattern. A 3-mm margin was added to each target volume to produce the following planning target volumes for the GTVnx, GTVnd, CTV1 and CTV2: PGTVnx, PGTVnd, PCTV1, and PCTV2, which received total radiation doses of 68–74 Gy, 60–71 Gy, 60–70.4 Gy, and 54–60 Gy, respectively, delivered in 30–32 fractions at five fractions per week over a period of 6~7 weeks. For concurrent chemotherapy, patients received a single-drug platinum-based regimen every 3 weeks for 2–3 cycles. All patients received cisplatin alone as the concomitant chemotherapy regimen. AC was administered to patents 28 days after CCRT. The AC schedule consisted of a combination of a platinum-based regimen with two or three drugs every 4 weeks for 2–3 cycles. In all, 304/334 patients (91.0%) received cisplatin and 5-fluorouracil, 28/334 patients (8.4%) received cisplatin and docetaxel, and 2/334 patients (0.6%) received cisplatin, 5-fluorouracil and docetaxel.

### Follow-up

After completion of the treatments, the conditions of the patients were examined every 3 months during the first 2 years, every 6 months in the third to fifth years, and yearly thereafter through clinic visits, written correspondence, or telephone interviews. The information obtained was used to evaluate patient survival, relapse patterns, and distant metastasis incidence. Examinations included ultrasound scans of the liver and abdomen, chest X-rays or computed tomography (CT) scans, CT or magnetic resonance imaging scans of the head and neck, whole-body bone scans, and nasopharyngoscopy with or without biopsy.

### Statistical analysis

All analyses were performed with SPSS software, version 16.0 (SPSS Inc, Chicago, IL). χ^2^ tests were utilised for comparisons of the distribution of selected factors and clinical characteristics. Significant differences in OS, DMFS, loco-regional relapse-free survival (LFFS), and failure-free survival (FFS) were estimated with the log-rank test. A multivariate analysis was performed with the Cox proportional hazards model to test the significance of independent prognostic factors. P-values ≤ 0.05 were considered statistically significant. Grouping by neutrophil count, platelet count, haemoglobin, serum alkaline phosphatase (ALP), serum ferritin, and serum lactate dehydrogenase (LDH) levels was performed using standards, whereas grouping by serum albumin levels was conducted according to to the methods of a published paper^9^. Three steps were used. First, the data from the 511 patients were used to identify the prognostic significance of pre-treatment clinical and laboratory factors for OS in univariate and multivariable analyses. Second, using independent prognostic factors, a prognostic score model for OS was generated. Each independent prognostic factor was integrated into one score. The cut-off score to distinguish the high- and low-risk groups regarding OS was determined using a receiver-operating characteristic (ROC) curve analysis. Finally, a stratification survival analysis was performed. The efficacy of AC was evaluated for each stratification of the entire cohort dichotomised by each individual prognostic factor and the prognostic score model.

## Results

Participants were followed until December 2015. At the median follow-up time of 49.7 months (range, 2.0–102 months), the 5-year cumulative survival rates for the CCRT-alone group were as follows: OS, 77.7%; DMFS, 84.3%; LFFS, 89.4%; and FFS, 74.7%. The 5-year cumulative survival rates for the CCRT + AC group were as follows: OS, 82.2%; DMFS, 83.6%; LFFS, 90.6%; and FFS, 75.4%. No significant differences were observed between the CCRT-alone and CCRT + AC groups for any end-point.

According to the univariate analysis, age, T classification, N classification, and serum albumin levels were significant prognostic factors for OS in patients with loco-regionally advanced NPC who received CCRT alone or CCRT + AC. These factors were then used to conduct a multivariate analysis. After the multivariate analysis, age, T classification, N classification, and serum albumin levels were still identified as independent prognostic factors for OS (Table 2).

**Figure 1:**
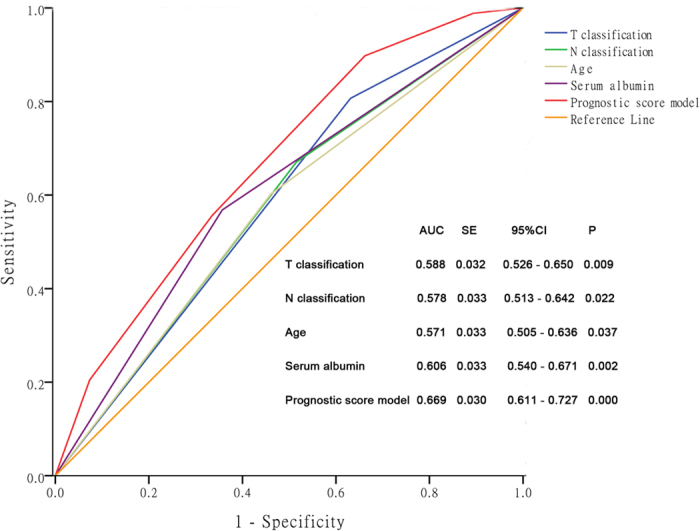
ROC curves for OS in loco-regionally advanced NPC patients who received concurrent chemoradiotherapy with or without AC based on the individual prognostic factors and a prognostic score model.

Using the four factors, we established a prognostic score model for OS in patients who received CCRT alone or CCRT + AC. Patients were sub-grouped by age, T classification, N classification, and serum albumin level. Each risk factor was integrated into one score. The ROC curves are shown in Fig. 1. The area under the curve for the prognostic score model was 0.669. A score of 1.5 resulted in a sensitivity of 0.898 and specificity of 0.338 for OS.

**Figure 2:**
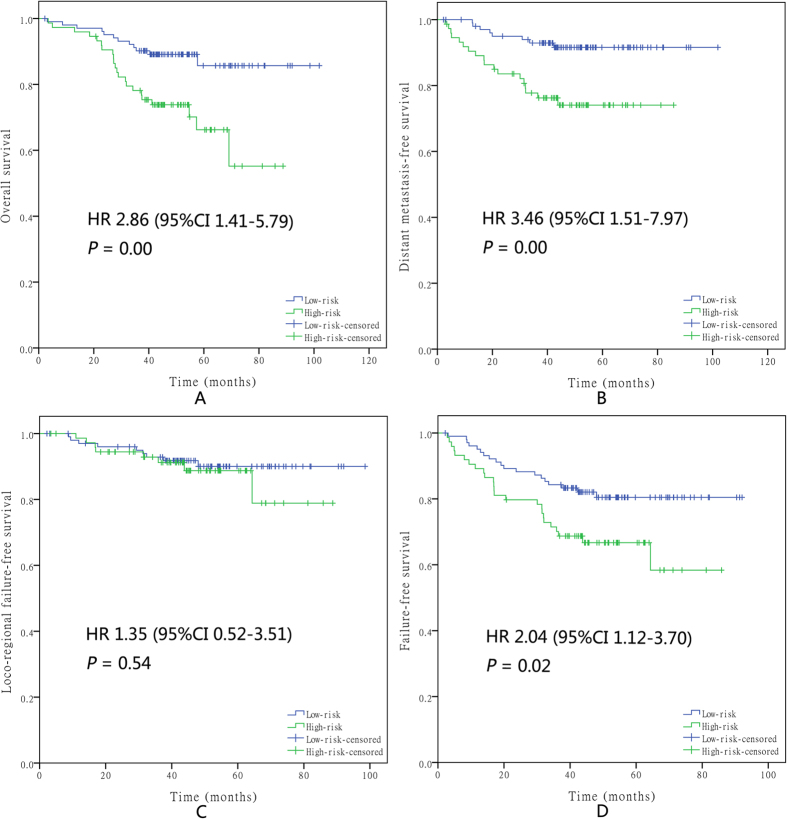
Kaplan–Meier survival curves for OS (**A**), DMFS (**B**), LFFS (**C**), and FFS (**D**) in the high- and low-risk groups of NPC patients who received CCRT alone. Every curve represents censored and uncensored data, and “+” represents censored data.

**Figure 3:**
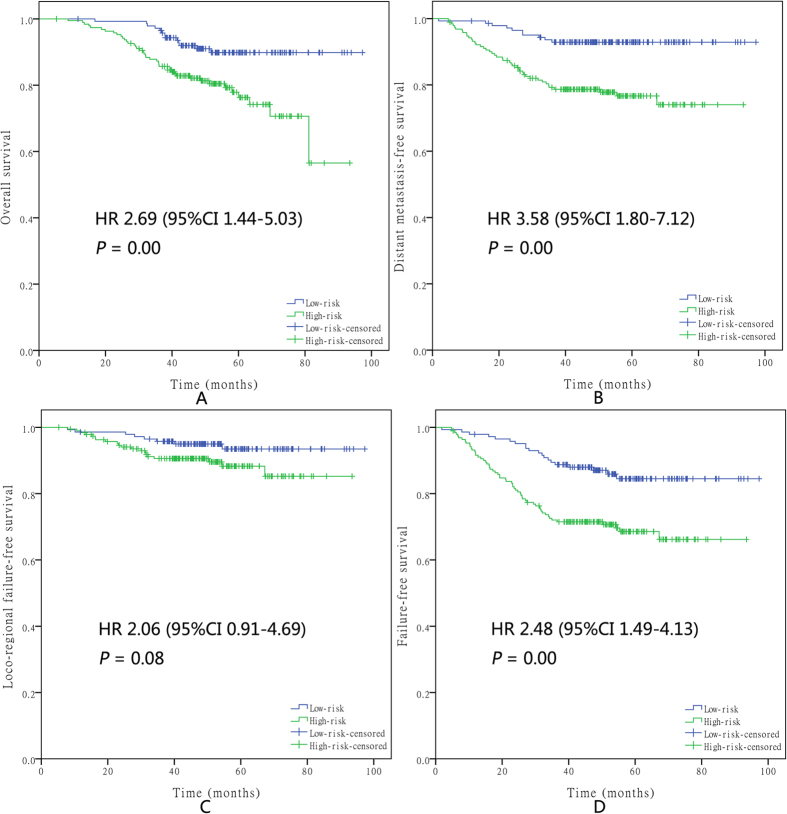
Kaplan–Meier survival curves for OS (**A**), DMFS (**B**), LFFS (**C**), and FFS (**D**) in the high- and low-risk groups of NPC patients who received CCRT + AC. Every curve represents censored and uncensored data, and “+” represents censored data.

Then, two risk stratification groups were generated: (1) low-risk: total score of 0–1 (152 patients); and (2) high-risk: total score of 2–4 (359 patients). For the CCRT-alone group, the survival analysis showed significant differences between the high- and low-risk groups regarding OS (hazard ratio (HR), 2.86; 95% confidence interval (CI) 1.41–5.79; P = 0.00), DMFS (HR, 3.46; 95% CI, 1.51–7.97; P = 0.00), and FFS (HR, 2.04; 95% CI, 1.12–3.70; P = 0.02) but not LFFS (HR, 1.35; 95% CI, 0.52–3.51; P = 0.54) (Fig. 2). For the CCRT + AC group, the survival analysis showed significant differences between the high- and low-risk groups regarding OS (HR, 2.69; 95% CI, 1.44–5.03; P = 0.00), DMFS (HR, 3.58; 95% CI, 1.80–7.12; P = 0.00), and FFS (HR, 2.48; 95% CI, 1.49–4.13; P = 0.00) but not LFFS (HR, 2.06; 95% CI, 0.91–4.69; P = 0.08) (Fig. 3).

**Figure 4:**
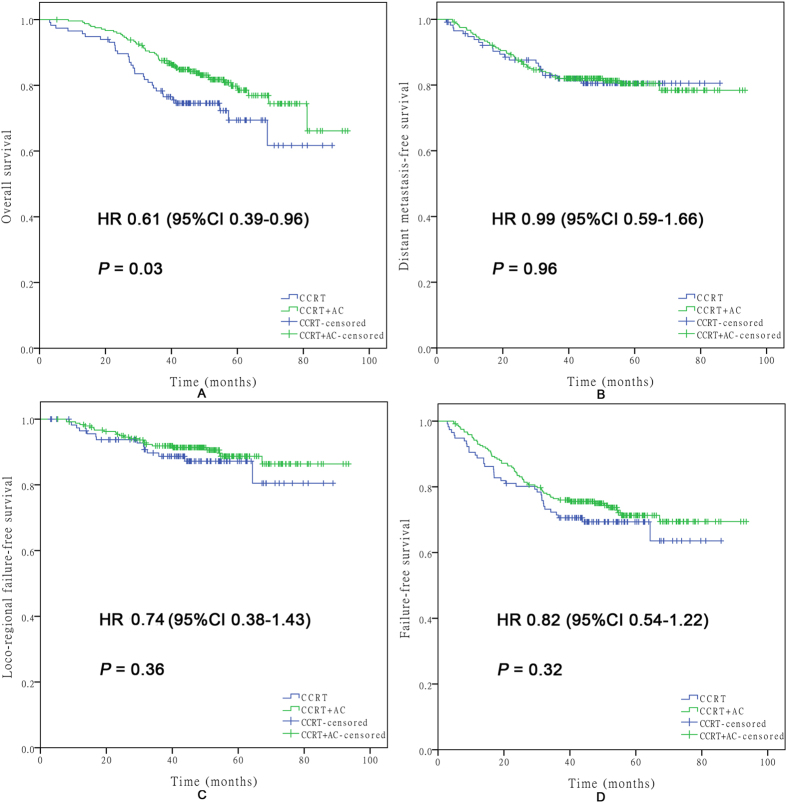
Kaplan–Meier survival curves for OS (**A**), DMFS (**B**), LFFS (**C**), and FFS (**D**) in the CCRT + AC and CCRT-alone groups of high-risk patients. Every curve represents censored and uncensored data, and “+” represents censored data.

**Figure 5:**
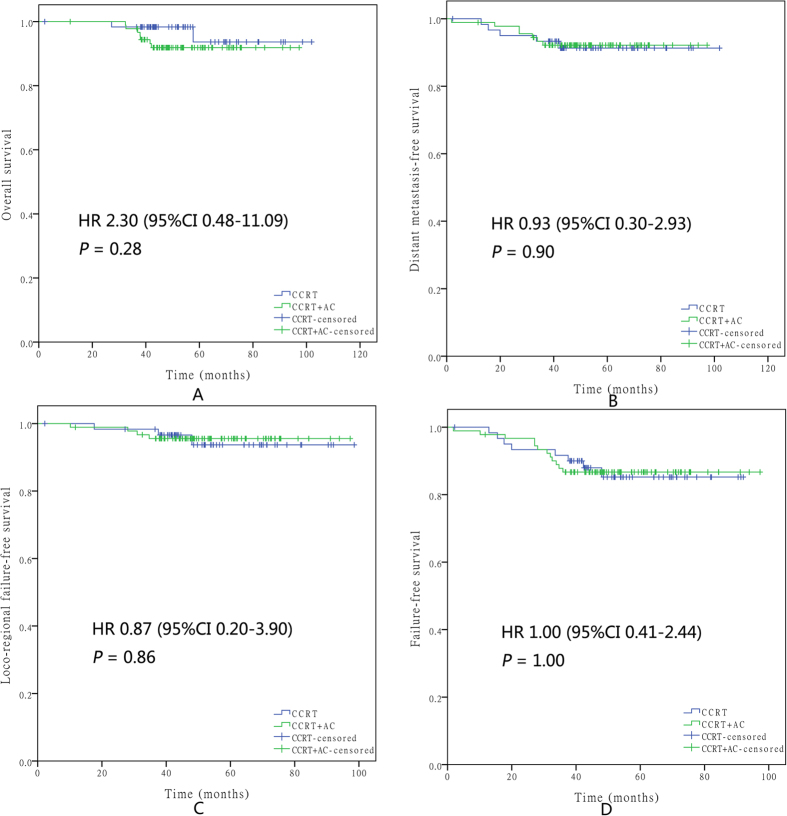
Kaplan–Meier survival curves for OS (**A**), DMFS (**B**), LFFS (**C**), and FFS (**D**) in the CCRT + AC and CCRT-alone groups of low-risk patients. Every curve represents censored and uncensored data, and “+” represents censored data.

A stratification survival analysis of the CCRT + AC group versus the CCRT-alone group was conducted according to age, T classification, N classification, serum albumin level, and the prognostic score model (Table 3). Within the high-risk group, the CCRT + AC group had a significantly higher OS than the CCRT-alone group (HR, 0.61; 95% CI, 0.39–0.96; P = 0.03). However, no significant differences were found in DMFS (HR, 0.99; 95% CI, 0.59–1.66; P = 0.96), LFFS (HR, 0.74; 95% CI, 0.38–1.43; P = 0.36), or FFS (HR, 0.82; 95% CI, 0.54–1.22; P = 0.32) between the two groups (Fig. 4). For the low-risk group, no significant differences were found in OS (HR, 2.30; 95% CI, 0.48–11.09; P = 0.28), DMFS (HR, 0.93; 95% CI, 0.30–2.93; P = 0.90), LFFS (HR, 0.87; 95% CI, 0.20–3.90; P = 0.86), or FFS (HR, 1.00; 95% CI, 0.41–2.44; P = 1.00) between the two groups (Fig. 5). Moreover, significant differences in OS were found for patients with T3-T4 stage disease (HR, 0.61; 95% CI, 0.38–0.99; P = 0.04) between the CCRT + AC and CCRT-alone groups.

## Discussion

To our knowledge, this may be the first study to establish a prognostic score model for selecting patients with loco-regionally advanced NPC who may gain a survival benefit from the application of AC after concurrent chemotherapy with IMRT. The present study showed that age, T classification, N classification, and serum albumin levels were significant prognostic factors for OS. Additionally, patients with two or more high-risk factors may benefit from the addition of AC to CCRT regarding OS, but not regarding DMFS, LFFS, or FFS.

The T and N stages often reflect the tumour burden, which has been reported to be associated with the prognoses of NPC^10^,^11^,^12^. Zong *et al*.^10^ performed a study to evaluate the 7th edition of the American Joint Committee on Cancer staging system for NPC. They found that the HRs for disease-specific survival and OS differed significantly between T2 and T3 and between T3 and T4. Pan *et al*.^11^ also reported that the T classification was an important prognostic factor for NPC. In 2016, Zhang *et al*.^12^ conducted a study to establish an integrated model incorporating the standard uptake value and N classification to predict metastasis in NPC. The data from 449 patients with stage I-IVB NPC who were treated with radiotherapy or chemoradiotherapy were retrospectively analysed. The results showed a significant difference in DMFS between patients with N2-N3 stage disease and those with N0-N1 stage disease (HR, 2.570; 95% CI, 1.422–4.579; P = 0.001). Wu *et al*.^13^ performed a retrospective study to explore tumour regression and patterns of distant metastasis in T1-T2 stage NPC treated with IMRT. They found that the distant metastasis rate was significantly higher in N2-N3 stage patients than in N0-N1 stage patients. In a trial conducted by Xiao *et al*.^14^, 229 NPC patients were analysed to determine the influence of gender and age on survival. The study showed that patients >45 years old had a significantly poorer 5-year OS than those ≤45 years old. A previous study by our group also showed that age was a significant prognostic factor and that 45 years of age was the cut-off point^15^. The serum albumin level was used as an index for evaluating the pre-treatment nutritional status. Li *et al*.^9^ demonstrated that a pre-treatment serum albumin level <43.0 g/L was related to poorer OS and DMFS (P < 0.001 and P = 0.042, respectively). In a trial by Du *et al*.^16^, the pre-treatment serum albumin level was an independent prognostic factor for distant metastasis in patients who received CCRT alone.

Chen *et al*.^2^ conducted a multicentre RCT to compare CCRT + AC versus CCRT alone in patients with loco-regionally advanced NPC. Zhang *et al*.^17^ performed a retrospective study to evaluate the value of AC after CCRT for the treatment of NPC. Neither study found a significant benefit in NPC from the addition of AC to CCRT. The trial by Chen *et al*.^2^ included 140/508 patients (27.6%) with T1-T2 stage disease and 156/508 patients (30.7%) with N1 stage disease. In the trial by Zhang *et al*.^17^, 63/189 patients (33.3%) exhibited stage II disease. These factors may be associated with the negative results. In a trial by Liang *et al*.^8^, the data from 260 patients with locally advanced NPC were analysed to determine whether the addition of AC after CCRT was necessary. After a mean follow-up time of 42.1 months, a borderline significant benefit was found for patients with N2-N3 stage disease (HR, 0.35; 95% CI, 0.11–1.06; P = 0.052). Therefore, for certain patients with loco-regionally advanced NPC, the addition of AC after CCRT may be essential. The present study showed that age >45 years old, T3-T4 stage, N2-N3 stage and serum albumin levels ≤42 g/L were significant independent prognostic factors for OS. In addition, a prognostic score model based on high-risk factors for OS was constructed. High-risk patients may exhibit improved OS with the addition of AC to CCRT. Compared with the high-risk patients, the low-risk patients have lower risks for death or distant metastasis; as such, they might not need to receive the additional AC. These findings may help oncologists select therapeutic regimens for patients with loco-regionally advanced NPC.

Our study has several limitations. First, as a retrospective study, selection bias may have occurred because patients were included only if they met specific selection criteria. Second, the sample size was not large. Third, recent studies have shown that the maximum standard uptake value obtained by ^18^F-fluorodeoxyglucose positron-emission tomography^12^, plasma Epstein–Barr viral DNA^18^,^19^, and the nutritional index^20^ are independent prognostic factors for NPC. However, not all patients have these relevant data. Therefore, these factors were not incorporated into the analyses in the present study. Finally, there were three AC regimens in the present study, which may have influenced the results. However, 304/334 patients (91.0%) received cisplatin and 5-fluorouracil; thus, the effect of the various regimens might have been negligible.

In conclusion, according to the prognostic score model based on age, T classification, N classification, and serum albumin, high-risk patients with loco-regionally advanced NPC may gain a survival benefit from the addition of AC after CCRT with IMRT. Furthermore, it is essential to conduct multicentre RCTs to determine whether the CCRT + AC regimen is superior to the CCRT-alone regimen in loco-regionally advanced NPC patients with two or more risk factors.

## Additional Information

**How to cite this article**: Liang, Z.-G. *et al*. Significant survival benefit of adjuvant chemotherapy after concurrent chemoradiotherapy in locally advanced high-risk nasopharyngeal carcinoma. *Sci. Rep.* **7**, 41449; doi: 10.1038/srep41449 (2017).

**Publisher's note:** Springer Nature remains neutral with regard to jurisdictional claims in published maps and institutional affiliations.

## Figures and Tables

**Table 1:** Characteristics of the 511 patients with NPC in the CCRT-alone and CCRT + AC groups.

Characteristics	CCRT alone (N = 177)	CCRT + AC (N = 334)	*P*
Sex			
male	137 (77.4%)	258 (77.2%)	0.97
female	40 (22.6%)	76 (22.8%)	
Age, years			0.02
≤45	78 (44.1%)	185 (55.4%)	
>45	99 (55.9%)	149 (44.6%)	
T classification*			0.00
T1-2	81 (45.8%)	92 (27.5%)	
T3-4	96 (54.2%)	242 (72.5%)	
N classification*			0.00
N0-1	100 (56.5%)	134 (40.1%)	
N2-3	77 (43.5%)	200 (59.9%)	
Neutrophil, k/cc			0.88
≤7	167 (94.4%)	314 (94.0%)	
>7	10 (5.6%)	20 (6.0%)	
Hemoglobin, g/L			0.07
≤120	27 (15.3%)	33 (9.9%)	
>120	150 (84.7%)	301 (90.1%)	
Platelets, k/cc			0.54
≤300	151 (85.3%)	278 (83.2%)	
>300	26 (14.7%)	56 (16.8%)	
Serum albumin, g/L			0.52
≤42	73 (41.2%)	128 (38.3%)	
>42	104 (58.8%)	206 (61.7%)	
Serum alkaline phosphatase, U/L			0.41
≤40	16 (9.0%)	38 (11.4%)	
>40	161 (91.0%)	296 (88.6%)	
Serum lactate dehydrogenase, U/L			0.03
≤245	171 (96.6%)	306 (91.6%)	
>245	6 (3.4%)	28 (8.4%)	
Serum ferritin, U/L			0.81
≤300	99 (55.9%)	183 (54.8%)	
>300	78 (44.1%)	151 (45.2%)	

Notes: NPC:Nasopharyngeal carcinoma; CCRT: Concurrent chemotherapy; AC: Adjuvant chemotherapy.

^*^AJCC: American Joint Committee on Cancer.

**Table 2:** Analysis of risk factors for NPC patient deaths in the CCRT + AC group and CCRT-alone group.

Factors	Univariate analysis		Multivariate analysis	
	HR (95% CI)	*P*	HR (95% CI)	*P*
Gender (Male vs Female)	1.30 (0.77–2.20)	0.33		
Age, years (>45 vs ≤ 45)	1.80 (1.17–2.76)	0.01	1.61 (1.03–2.50)	0.04
T stage (T3-4 vs T1-2)	2.47 (1.45–4.20)	0.00	2.05 (1.20–3.51)	0.01
N stage (N2-3 vs N0-1)	1.84 (1.18–2.88)	0.00	1.84 (1.17–2.89)	0.01
Leukocytes, k/cc (>7 vs ≤ 7)	1.89 (0.95–3.77)	0.07		
Hemoglobin, g/L (>120 vs ≤ 120)	0.90 (0.50–1.66)	0.74		
Platelets, k/cc (>300 vs ≤ 300)	1.30 (0.76–2.20)	0.34		
Serum albumin, g/L (>42 vs ≤ 42)	0.46 (0.30–0.70)	0.00	0.56 (0.36–0.86)	0.01
Alkaline phosphatase, U/L (>40 vs ≤ 40)	2.26 (0.92–5.58)	0.07		
Lactate dehydrogenase, U/L (>245 vs ≤ 245)	1.45 (0.70–3.00)	0.31		
Serum ferritin, mg/L (>300 vs ≤ 300)	1.34 (0.89–2.04)	0.16		

Notes: NPC: Nasopharyngeal carcinoma; CCRT: Concurrent chemotherapy; AC: Adjuvant chemotherapy; HR: Hazard ratio.

**Table 3:** Stratification survival analysis of the CCRT + AC group versus the CCRT-alone group for all patients.

	OS		DMFS		LFFS		FFS	
Subgroups	HR (95% CI)	*P*	HR (95% CI)	*P*	HR (95% CI)	*P*	HR (95% CI)	*P*
**Age**, **years**								
≤45	1.44 (0.65–3.18)	0.37	1.09 (0.55–2.20)	0.79	1.59 (0.53–4.80)	0.40	1.21 (0.66–2.22)	0.55
>45	0.62 (0.36–1.06)	0.08	1.03 (0.54–1.96)	0.93	0.59 (0.27–1.28)	0.18	0.78 (0.49–1.27)	0.32
T classification*								
T1-2	0.91 (0.35–2.35)	0.84	1.11 (0.41–2.99)	0.83	0.62 (0.22–1.79)	0.37	0.85 (0.40–1.78)	0.66
T3-4	0.61 (0.38–0.99)	0.04	0.85 (0.50–1.46)	0.56	0.88 (0.40–1.90)	0.74	0.78 (0.51–1.21)	0.26
N classification*								
N0-1	0.63 (0.30–1.31)	0.21	0.77 (0.33–1.81)	0.55	0.53 (0.20–1.42)	0.20	0.56 (0.30–1.04)	0.06
N2-3	0.76 (0.44–1.30)	0.31	0.99 (0.56–1.76)	0.97	0.98 (0.43–2.21)	0.96	1.04 (0.64–1.69)	0.88
**Serum albumin**, **g**/**L**								
≤42	0.73 (0.42–1.29)	0.28	0.99 (0.53–1.86)	0.98	0.83 (0.36–1.93)	0.67	0.87 (0.52–1.45)	0.58
>42	0.90 (0.46–1.76)	0.76	1.15 (0.57–2.33)	0.69	0.83 (0.35–1.97)	0.67	0.98 (0.57–1.67)	0.93
**Serum alkaline phosphatase**, **U**/**L**								
≤40	0.56 (0.09–3.37)	0.52	0.80 (0.07–8.78)	0.85	0.59 (0.23–1.53)	0.27	0.76 (0.14–4.18)	0.76
>40	0.81 (0.52–1.26)	0.34	1.08 (0.67–1.74)	0.76	0.90 (0.40–2.05)	0.81	0.93 (0.64–1.36)	0.71
**Prognostic score model**								
Low risk (score 0–1, n = 152)	2.30 (0.48–11.09)	0.28	0.93 (0.30–2.93)	0.90	0.87 (0.20–3.90)	0.86	1.00 (0.41–2.44)	1.00
High risk (score 2–4, n = 359)	0.61 (0.39–0.96)	0.03	0.99 (0.59–1.66)	0.96	0.74 (0.38–1.43)	0.36	0.82 (0.54–1.22)	0.32

Notes: NPC: Nasopharyngeal carcinoma; CCRT: Concurrent chemotherapy; AC: Adjuvant chemotherapy; OS: Overall survival; DMFS: Distant metastasis-free survival; LFFS: Loco-regional failure-free survival; FFS: Failure-free survival; HR: Hazard ratio.

^*^AJCC: American Joint Committee on Cancer.
